# The Potential of Dietary Antioxidants

**DOI:** 10.3390/antiox10111752

**Published:** 2021-11-01

**Authors:** Irene Dini

**Affiliations:** Department of Pharmacy, University of Naples Federico II, Via Domenico Montesano 49, 80131 Napoli, Italy; irdini@unina.it

Oxidative stress happens when the levels of reactive species made from oxygen and nitrogen exceed the body’s antioxidant capacity. Exogenous agents (i.e., UV rays and ionizing radiation) and endogenous processes (i.e., incomplete reduction of O_2_ to H_2_O in oxidative phosphorylation reactions, inflammation, and infections) [1] generate reactive nitrogen species (RNS), including nitric oxide (^•^NO), and reactive oxygen species (ROS), including superoxide (O_2_^•−)^, hydroxyl radical (^•^OH), hydrogen peroxide (H_2_O_2_), singlet oxygen, and ozone, which can produce oxidative stress. The regulation of ROS and RNS levels is indispensable to preserve cellular homeostasis. Low ROS and RNS levels stimulate immune responses, cell proliferation, apoptosis, differentiation, and stress-responsive pathways. High ROS and RNS levels damage lipids, proteins, and DNA (breaking single- or double-strands, modifying base, and DNA cross-links) [2]. Oxidative stress causes chronic diseases such as cardiovascular diseases, cancer, Alzheimer, chronic obstructive pulmonary, and neurodegenerative pathologies [3]. Antioxidant systems defend human cells from free radicals. They act by stopping free radicals, decreasing their development, and quenching the formed ROS and RNS [4]. Some enzymes are involved in the antioxidant defense system; among these, glutathione (GSHs) (reductase, peroxidases, and *S*-transferases) can neutralize ROS directly or with the help of metal cofactors (e.g., Mn, Cu, Se, and Zn) [5]. The antioxidant molecules are classified into primary and secondary defense molecules. The primary antioxidant molecules (i.e., vitamins C and E, ubiquinone, and glutathione) reduce oxidation effects by moving a proton to the free radical species or electron donors, or by terminating the chain reactions [6]. The secondary antioxidants (i.e., *N*-acetyl cysteine and lipoic acid) act as cofactors for some enzyme systems or neutralize the production of free radicals by transition metals [7]. In recent years, the consumption of foods rich in nutrients and phytochemicals (secondary plant metabolites) with antioxidant properties has been linked to positive health outcomes [2,8,9,10]. Food supplements containing natural antioxidant molecules have been formulated to satisfy the consumers’ attention towards products containing natural molecules considered non-toxic, capable of preventing the pathologies related to oxidative stress, or improving skin and hair wellness [5]. Moreover, antioxidant molecules have been added to packaging materials to preserve food from oxidation and microorganism attack [11]. Unfortunately, in the plant kingdom, although the concentrations of antioxidants are widespread, they are low. Therefore, to increase the sources of antioxidant raw materials, it is necessary to improve the following:

1. Knowledge on the plants that express the greatest concentration of antioxidant molecules of interest,
2. Pedoclimatic conditions and cultivation stages that make most of their levels available in plants,
3. Extraction methods that can properly maximize their recovery,
4. The potential preventive and/or therapeutic effect of each chemical class of antioxidants,
5. Pharmaceutical delivery systems that allow for the full advantage of their actions in the body.

This work comprises original research papers and reviews on antioxidant molecules in food, the agricultural practices that maximize their levels in plants, the potential preventive effects of selected classes of antioxidant molecules, their potential use in functional foods, and the pharmaceutical delivery systems that maximize their potential activity when used as supplements. Finally, the recovery of food antioxidants from food waste is discussed, considering the need to reduce waste to protect the environment as an issue of primary importance [12].
